# Impactful educational transitions: Crossroads for physiotherapy education in South Africa?

**DOI:** 10.4102/sajp.v78i1.1638

**Published:** 2022-04-29

**Authors:** Corlia Janse van Vuuren

**Affiliations:** 1 School of Health and Rehabilitation Sciences, Faculty of Health Sciences, University of the Free State, Bloemfontein, South Africa

**Keywords:** Doctor of Physical Therapy, physiotherapy technician, community health worker, National Health Insurance, physiotherapy education, Agenda 2063, National Development Plan 2030, Sustainable Development Goals

## Abstract

**Background:**

Global changes in physiotherapy entry-level educational programmes to exit with a Doctorate or Master’s degree have consequences if physiotherapy education, worldwide, is to remain professionally competitive. However, within the South African context, such global competitiveness should be carefully considered against the national healthcare needs and implementation of the National Health Insurance (NHI) programme, with a bigger emphasis on a skilled mid-level workforce, including physiotherapy technicians or community rehabilitation workers.

**Objectives:**

These competing interests are carefully considered, against the theoretical background of international DPT training; human resource and financial constraints in the South African healthcare sector; reforms of the South African health and educational sectors intended to address the inequalities of the past; the need for quality healthcare delivery and the professional reputation of physiotherapy in South Africa.

**Methods:**

A framework for physiotherapy education in South Africa, to move on from the current educational crossroads, is proposed through an integration of multiple theoretical perspectives.

**Results:**

The framework is based on the current challenges being experienced in physiotherapy education and healthcare service delivery, which could be addressed by changes in the education sphere.

**Conclusion:**

The baseline suggestions for (re)considering the current education environment for physiotherapy, as proposed in my article, are to ensure that the profession remains relevant and able to confront the current changes presented by the South African healthcare system, including the implementation of the NHI plan, whilst remaining globally aligned and competitive.

**Clinical implications:**

The suggested, reconsidered, educational framework for physiotherapy in South Africa could become pivotal in advancing the profession on both a national and international level, through further critical conversations.

## Introduction

Current debates within the physiotherapy profession are driven by international changes in physiotherapy education, such as the transition to Doctor of Physiotherapy/Physical Therapy (DPT) exit-level programmes in the United States, as well as local needs to improve healthcare through the implementation of the National Health Insurance (NHI). Some physiotherapists advocate for the introduction of an academic programme for the continued training of physiotherapy technicians (PTTs), or even the training of multi-professional community rehabilitation workers (CRWs), whilst others support changing the current professional physiotherapy training programme to a DPT programme. These conflicting suggestions need careful consideration if sound decisions are to be made to enable physiotherapy education in South Africa to advance in the right direction from this educational crossroad, as such educational decisions could have far-reaching consequences for the profession. On the one hand, educational decisions should be directed towards addressing the healthcare needs of the country sufficiently; on the other hand, the profession needs to become even more reputable nationally whilst remaining competitive and internationally recognised.

## Background and literature review

Several key national and international directives inform the positioning of physiotherapy and physiotherapy education in South Africa. On a national level, the National Development Plan 2030, the NHI implementation and the policies within the South African education system currently play a pivotal role. Internationally, the profession is guided by the Sustainable Development Goals (SDGs), the African Union Commission’s Agenda 2063, the World Health Organisation’s Vision for Primary Health Care in the 21st century and the view of *World Physiotherapy* on the future of physiotherapy training. In this literature review, each of these directives is briefly unpacked and linked to physiotherapy education (specifically in South Africa) to sketch the context in which the educational future of physiotherapy in South Africa is (re)considered in my article.

### National Development Plan 2030 and National Health Insurance in South Africa

South Africa has seen many changes since the end of the apartheid regime in 1994, and a major emphasis on the development and reform of the health and educational sectors are two of the focal points to address the inequalities of the past. The National Development Plan 2030 highlights the slow progress in these areas since 1994 and identifies the inability of the public health system to meet the demand or sustain the quality as one of the key challenges (RSA NPC 2011:15). To address the challenges facing the health sector, the introduction of the NHI system, and the provision of ‘more and better-trained health professionals’ is proposed (RSA NPC 2011:41–42).

However, to move to a universal healthcare system (such as the NHI), a ‘skilled, enabled and supported health workforce’ will be needed (RSA DoH 2020:2). The experiences of comparable countries, such as Brazil, Ghana and Mexico, have underlined the importance of investing in the healthcare workforce. Besides improved health outcomes, an investment in the healthcare workforce itself also drives inclusive economic growth and job creation (RSA DoH 2020:2). Although South Africa, currently, has a higher healthcare professional-to-patient ratio than most other African countries, it is anticipated that an additional 97 000 healthcare workers (of which one third should be community healthcare workers [CHWs]) are required to improve access to quality healthcare and equality in the country (RSA DoH 2020:19). With this clear need for additional, skilled healthcare workers to implement the NHI successfully, it is important for the physiotherapy profession to consider its professional profile and to continue to address the inequalities of the past through informed educational decisions and the implementation of sound educational transitions. A review by Louw et al. (2020) highlighted that transformation in the profile of physiotherapists is not optimal and that educational transitions could play an important role by becoming more actively responsible for transformation in the profession. The inclusion of training of PTTs or CRWs could thus not only address the human resource needs for the implementation of the NHI but also assist with addressing the much-needed transformation in the profession. Such educational transitions should, however, have clearly articulated options at different levels to make them viable, as proposed later in my article.

Additionally, the implementation of the NHI *benefits package* would require a further 88 000 primary healthcare (PHC) workers by 2025 (RSA DoH 2020:19). When appointing additional healthcare workers for the NHI, the current distribution (and inclusion) of professions, the needs per level of care and the required skills mix should be considered carefully (RSA DoH 2020:20). Educational transitions within professions (such as physiotherapy) should thus be based on the full skills-set required for the NHI, whilst considering the existing human and financial constraints in the public health sector. Ross, Gumede & Mianda (2018) note that the biggest challenge in the public sector is poor long-term retention, resulting in most of the workforce being young and transient. This could have a negative effect on the skills mix of the workforce and could limit the positive outcomes foreseen for the NHI. Some strategies to temporarily alleviate human resource constraints in the public healthcare sector have included the introduction of compulsory community service in professions such as physiotherapy (Nadasan & Chetty 2020), as well as extended or decentralised clinical training implemented by universities such as the University of Kwazulu-Natal (Govender et al. 2018).

Based on the expressed need for an expanded and skilled healthcare workforce in South Africa (as discussed above), the critical decision for professions (such as physiotherapy) is, thus, how to position themselves to be reputable enough to be included as a vital component in the aforementioned *benefits package* of the NHI whilst also being ready to provide the (extended range of) human resources and the skills mix that might be required for the NHI system in South Africa. Even though both the suggested options, namely PTT/CRW training and DPT training, present valuable arguments for this scenario, the author would like to propose a possible integration of the two options, as outlined later in this article, as the most viable option to position physiotherapy education in the best way possible.

### Healthcare education in South Africa

The education sector recognises the health sector’s NHI objectives and need for, amongst others, extended human resources, and reacted by introducing a 240-credit diploma option in the Higher Education Qualifications Sub-framework (HEQSF) (CHE 2013:25). The inclusion of this exit-level option in the HEQSF allows (within the professional pathway) for an appropriate level training for mid-level health professionals. A second advantage of this profession-specific option in the HEQSF is that it can provide a basis for such health professionals to work towards a professional Bachelor’s degree, and beyond, in that profession (CHE 2013:25). The HEQSF clearly alludes to the role of statutory bodies (such as the Health Professions Council of South Africa [HPCSA]) in the accreditation and recognition of these profession-specific qualifications to register these professionals for healthcare practice in South Africa (CHE 2013:15).

In the context of the NHI and the anticipated increase of the healthcare workforce, especially at the PHC level, as mentioned above, the training of PTTs/CRWs seems to hold value. The evaluation of the first phase of NHI implementation in different pilot districts found that the ward-based PHC outreach teams (WBPHCOTs) were a ‘critical programme that should be continued and strengthened’ (Genesis Analytics 2019:17). Some of the recommendations, going into Phase 2 of the NHI implementation, have evolved from the biggest challenges experienced by the WBPHCOT element in Phase 1 of the NHI implementation. These include that WBPHCOTs need regular and appropriate supervision; that the scope of practice of CHWs should be reviewed and possibly expanded in the areas of health promotion and referrals and that electronic systems would enable teams to report on household data (Genesis Analytics 2019:17). Myezwa and Van Niekerk (2013:4) refer to the inclusion, in the NHI, of rehabilitation services at the district hospital level and also list underfunding and pooled resources as considerations when negotiating the inclusion of rehabilitation at different levels of care. With the recommendations from Phase 2 of the NHI referring to an expansion in the scope of practice of CHWs, to focus on health promotion and referrals (Genesis Analytics 2019:17), clear evidence of the role physiotherapists could play in the realm of health promotion exists (Narain & Mathye 2019), and there might be an opportunity to utilise PTTs/CRWs in this area. Not only could PTTs/CRWs add considerable value to the WBPHCOTs because of the former’s knowledge of health promotion, but PTTs/CRWs could also fulfil a pivotal role in the supervision of WBPHCOTs and create an effective referral system (especially at the district hospital level, where therapists will be available). This would support the view of Myezwa and Van Niekerk (2013:8) that rehabilitation professionals should, perhaps, respond to universal health coverage (or the NHI) by offering a service that is not necessarily profession-based, but more holistic. Training of PTTs/CRWs within the HEQSF’s provision of a 240-credit programme could be tailor made for this purpose. The availability of different education opportunities within the HEQSF, once again, emphasises how important it is for professions, including physiotherapy, to position themselves adequately within the changing health and education systems.

### Sustainable Development Goals, Agenda 2063 and the World Health Organization’s vision for primary health care

**FIGURE 1:**
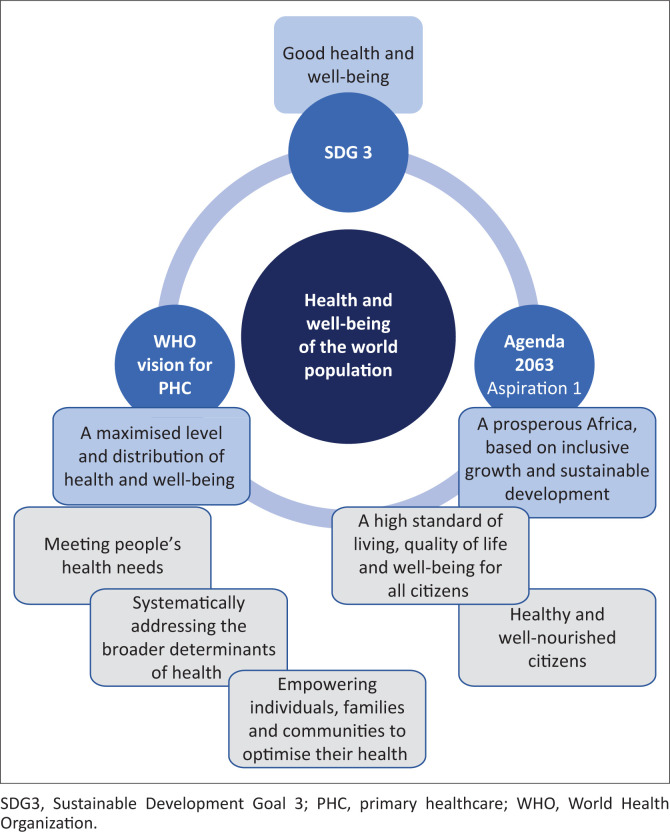
Alignment of SDG 3, Agenda 2063 Aspiration 1 and the World Health Organisation vision for primary healthcare in the 21st century.

The health and well-being of the world’s population remain the key focus of the United Nation’s SDGs and the African Union Commission’s Agenda 2063. With a clear alignment between, specifically, SDG 3 (United Nations 2015:20) and Aspiration 1 of Agenda 2063 (APRM 2020:33) (see Figure 1), South Africa could play a vital role in achieving ‘The Africa We Want’. The key SDG interventions, which should support the attainment of Africa 2063 Aspiration 1 within the health and well-being domain, are ‘universal health coverage [such as the NHI implementation in South Africa], healthy behaviours and social determinants of health and well-being’ (APRM 2020:46). Additionally, the alignment amongst SDG 3, universal healthcare access and PHC is highlighted in the World Health Organisation’s Vision for Primary Health Care in the 21st century (see Figure 1). Through this vision, PHC is viewed as

[*A*] whole-of-society approach to health that aims equitably to maximise the level and distribution of health and well-being by focusing on people’s needs and preferences (both as individuals and communities). (WHO & UNICEF 2018:xii)

Narain and Mathye (2019) recently highlighted the important role of physiotherapy in achieving the SDGs, specifically in the South African context. This also creates the opportunity to position physiotherapy and physiotherapy education centrally, within the ambit of Africa 2063. With an innovative and strategic reconsideration of the physiotherapy levels of care, the physiotherapy skills mix and the positioning of physiotherapy (and physiotherapy education) to support NHI implementation, as well as the attainment of the SDGs and Africa 2063, physiotherapy (as a profession) could remain pivotal in the South African and African health contexts. New possibilities for physiotherapy education in South Africa, to support the anticipated future of healthcare in South Africa (and Africa), are presented later in my article. Such new educational transitions, or possibilities, should be built on the current educational principles of creating positive student–staff pedagogical relationships (Amosun et al. 2018; Ramklass & Vithal 2021), creating relevant curricula for the South African healthcare context (theoretical, practical and clinical) (Chetty et al. 2018) and decolonisation (Cobbing 2021). The importance of these aspects is acknowledged by the author, but an in-depth discussion of these principles is not included in my article, as it falls beyond the scope of my article, which is focusing on educational transitions.

### World physiotherapy’s view on global physiotherapy education

Physiotherapy education worldwide is guided by *World Physiotherapy*. The newly released physiotherapist education framework (World Physiotherapy 2021:5) provides clear guidelines on the development of physiotherapy education programmes, which need to be considered within the context of my article. Interestingly, reference is made to the use of the term ‘physiotherapy’ in titles of education programmes. The framework, therefore, refers to entry-level physiotherapy programmes that include physiotherapy in their title, and these are, at least, at a university Bachelor’s level, thereby ensuring that physiotherapists can practise independently and have developed the necessary ‘theoretical, cognitive, practical, professional, and transferable skills which underpin the practice of physiotherapy’ (World Physiotherapy 2021:24). In some countries, like South Africa, Canada and the United States, this naming convention has significant implications for physiotherapy assistant (PTA) and/or PTT designations and training, which contain the word physiotherapy in their titles, but are not at a Bachelor’s level. This should also be considered carefully in the context of reconsidering physiotherapy education in South Africa.

All over the world, physiotherapists are trained by means of 4-year Bachelor/Bachelor of Science degree programmes, including in South Africa. The main exceptions are the United States, which offers an entry-level Doctorate programme in physiotherapy (APTA 2018), and Canada, with an entry-level Master’s degree in physiotherapy (Canadian Council of Physiotherapy University Programs 2021). Since the implementation in 2015 (graduating from 2018), the American Physical Therapy Association (APTA) only recognises a Doctor of Physical Therapy (DPT) degree as the **entry-level** physiotherapy training programme. Several transitional DPT (t-DPT) programmes have, since then, been implemented to allow physiotherapists to convert their qualifications to DPT (APTA 2020). The main reason for this change was to ensure that physiotherapists in the United States can practise as autonomous practitioners (New York University 2021). This more intensive, profession-specific training could enhance the quality of the service delivered by these physiotherapists in the healthcare system. However, there is also some opposition to this change; some believe that the use of the designation ‘doctor’ could be confusing to patients and might create problems in the healthcare system as patients might find it difficult to distinguish between different healthcare professionals (i.e. medical doctors, physiotherapists) attending to their medical needs (Harris 2011).

These international changes and differences highlight the current educational crossroads for physiotherapy education in South Africa, by adding questions relating to international comparability and recognition to the existing questions of national healthcare provision in an under-serviced and changing healthcare system. Within this complex situation, questions relating to professional reputation and titles, such as ‘doctor’ in the United States, could be added to the debate.

## Towards a South African physiotherapy education landscape with multiple goals

Changes in the national health and education sectors, as well as changes in the international training of physiotherapists, should be considered carefully, to enable all stakeholders in South Africa to take informed decisions and move physiotherapy education beyond the current crossroads through sound educational transitions. On the one hand, the needs of the healthcare sector in South Africa, specifically in relation to the implementation of the NHI, should be considered. The human resource needs for the implementation of the NHI (RSA DoH 2020), inclusive of the expected additional number of healthcare workers, the skills-mix needs, the needs per level of care and the distribution of healthcare workers to deliver the *benefit packages*, should be carefully considered against the current human and financial resource constraints within the public healthcare sector. Consideration should also be given to evidence related to rehabilitation services in the South African healthcare sector (Mlenzana et al. 2013), including reference to current services from CHWs and CRWs. It should further be noted that the change to a DPT degree in the United States was to enable physiotherapists to function as first-line practitioners. In South Africa, as in other countries such as Canada, physiotherapists already have first-line practitioner status (Diener 2010:2; SASP 2012:2) and could thus remain internationally compatible/comparable within the realms of the profession.

**FIGURE 2:**
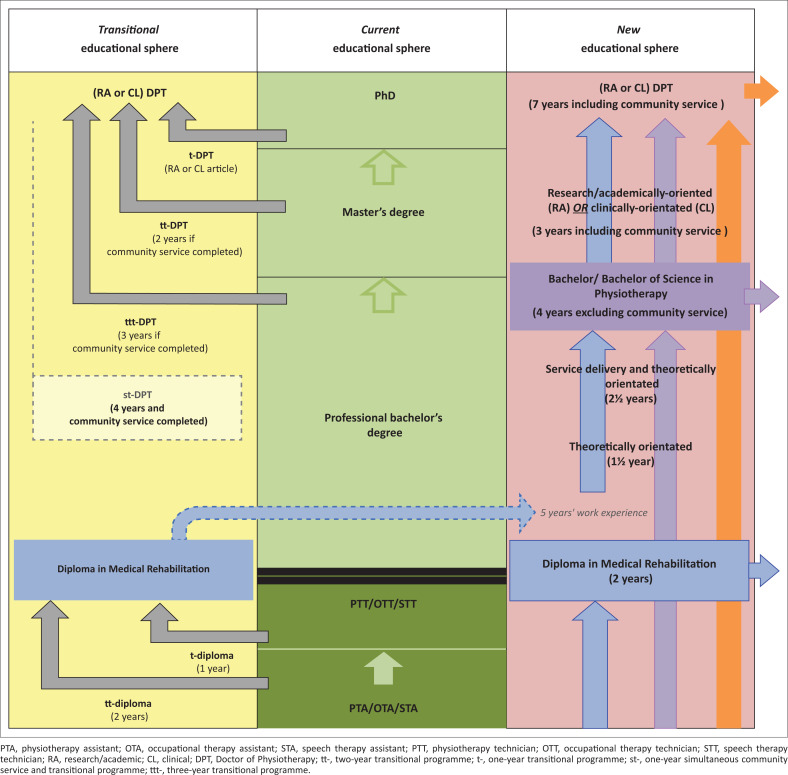
Framework for South African physiotherapy educational environment with multiple goals.

In Figure 2, the author suggests an integrative framework that could address many of the national and international issues that impact physiotherapy education in South Africa, as discussed in my article. This framework could act as a starting point for further discussion to move physiotherapy education in South Africa beyond the current crossroads it is facing. The proposed integrative framework attempts to expand the contribution of the physiotherapy profession at various service levels, as proposed by the NHI implementation plan and accompanying human resource plan (RSA DoH 2020), by means of an extended skills-mix whilst remaining internationally compatible/comparable and competitive. These aims are addressed by (1) the inclusion of PTAs/technicians (or some designation of rehabilitation technician) to serve underserved communities optimally at PHC levels; (2) positioning some physiotherapists as **clinical experts** at district and/or tertiary level, to provide high-quality knowledge and skills at more advanced levels of service delivery; (3) positioning other physiotherapists as **academic experts**, to provide expert research and academic competencies/input to the profession and (4) offering career advancement opportunities and transitional educational opportunities to previously qualified physiotherapists, nationally and internationally.

Figure 2 shows three clearly distinguishable spheres, namely the current education sphere (middle, green track), the proposed transitional educational sphere (left, yellow track) and the proposed, reconsidered educational sphere (right, pink track). The rest of the discussion will utilise the current (middle) educational sphere as the starting point, from where the transitional and proposed new spheres will be linked to each level of training.

Profession-based assistants and/or technicians (e.g. PTAs/PTTs, OTAs/OTTs) play a pivotal role in many healthcare systems, such as the national health systems in the United Kingdom, Australia and the United States, as well as in African countries, such as Malawi (Frantz 2007:3). Training of PTAs/PTTs, OTAs/OTTs, as well as community speech and hearing workers and speech (and hearing) therapy assistants, has also been implemented in South Africa, with subsequent registration with the respective HPCSA professional boards. However, training opportunities, job opportunities and career advancement opportunities have been limited for these individuals in the South African healthcare system, which suggests the need for a vital (re)consideration by policymakers. The value of this cadre of rehabilitation-based healthcare workers in South Africa is undisputed (Gamiet & Rowe 2019). However, a clear distinction should be made between CHWs (as appointed within the NHI plan of South Africa), and CRWs, as the scope of practice and/or focus of these two cadres of healthcare workers should, in my opinion, and as offered in my article, differ.

The main foci of CHWs within the NHI would be to assess the health status of individuals, to provide health promotion education, to **identify** people in need of preventive, curative or rehabilitative services and to refer those in need of services to the relevant PHC facility (RSA DoH 2015:32). Based on the challenges identified from Phase 1 of the NHI implementation (see above), introducing CRWs seems to be a viable option. Firstly, these CRWs could oversee the CHWs (instead of nurses doing so), within a predominantly nurse-driven health environment (RSA DoH 2020:20), which could relieve some of the current tensions identified in the NHI pilot review (Genesis Analytics 2019). Secondly, CRWs could add value through their extended skill mix by having a deeper understanding of health promotion and the referral of individuals to multiple healthcare professionals within a preventative, curative and rehabilitative healthcare environment. This arrangement would not only address the identified challenges facing the NHI and build positively on the work/requirements for CHWs within the NHI but would also address some of the extensive rehabilitation needs within communities (Gilmore et al. 2017).

In the new, suggested educational sphere (see Figure 2), the implementation of a Diploma in Medical Rehabilitation needs to be considered. This qualification would enhance the current profession-based assistant and/or technician space by (1) providing knowledgeable individuals, especially within the field of rehabilitation (cross-disciplinary), to support the implementation of the NHI and, ultimately, the extensive need for rehabilitation services within communities whilst supporting health promotion in communities and (2) providing graduates from this Diploma with greater mobility and even international opportunities through possible articulation to the Bachelor of Medical Rehabilitation programmes offered by a number of African universities (see earlier discussion), or profession-specific Bachelor programmes in South Africa. For PTAs, PTTs and other similar profession-specific assistants and technicians already registered with the HPCSA, a transitional educational pathway (see Figure 2) could be offered through either a two-year (tt-Diploma) or one-year (t-Diploma) programme. These transitional programmes could be superimposed on the newly suggested diploma programme to ensure that exit-level outcomes and standards are equitable. The diploma programme should be developed to address key national and global issues within the field of rehabilitation (including caregiver/family education), as well as health promotion. Furthermore, articulation to a profession-specific Bachelor’s programme (such as within physiotherapy or occupational therapy) could be provided after gaining a minimum of 5 years’ work experience within the NHI (or similar) system.

The next level of the educational framework (see Figure 2) deals with the current professional Bachelor’s degree in physiotherapy, also described as the entry-level physiotherapy qualification by *World Physiotherapy* (see earlier discussion). Possibly introducing a DPT programme as an exit-level programme (as currently in the United States) would be the area that affects most of the currently registered physiotherapists in South Africa. A great deal of attention will, thus, have to be directed to the transition of these physiotherapists to a DPT level whilst also considering other mobility or international opportunities (as alluded to earlier with the diploma programme). Within Figure 2, two articulation options are provided for these physiotherapists, depending on whether their community service has been completed or not. The reason for integrating community service as one of the transitional pathways (st-DPT) is to, firstly, address the current challenge faced by physiotherapists with qualifications from outside South Africa and/or international students in securing community service placements that would allow them access to the board examination and, ultimately, the opportunity to practise in South Africa and, secondly, to allow newly qualified students to transition into the DPT if implemented. It is important to note that the professional Bachelor or Bachelor of Science degree in physiotherapy is maintained as an exit-level programme in the new educational sphere (see Figure 2), thereby allowing for mobility in the opposite direction, namely to provide graduates with mobility and a level of internationalisation to pursue Master’s and/or transitional DPT studies abroad. As these graduates would not have completed their community service in South Africa, they will have to complete some form of transitional programme (see Figure 2), in case they wish to return to and practise in South Africa later. The st-DPT programme, as suggested in this article, could extend over 4 years, including community service or service in the South African PHC system, as well as a combination of further theoretical (academic) and research content. It is anticipated that the transition programme would focus on more dedicated fields of interest, grouped together, in which theory (academic) and research training would take place, for example, neurology, cardiopulmonary therapy, musculoskeletal therapy. As an additional layer, students would then be able to either follow a clinical focus (i.e. advanced clinical skills training and clinical research) or a research/academic focus (i.e. specific sub-area teaching and learning and educational research) (see Figure 2 for the distinction between a research/academic (RA) DPT and a clinical (CL) DPT). In my opinion, the distinction between RA and CL DPTs would enable the profession to position itself nationally and globally and to build on high-level, high-quality research, teaching and clinical skills. This distinction may also present opportunities for expert-based research and teaching between academics and clinicians and provide opportunities to narrow the significant knowledge-practice gap that currently exists (Baatiema 2018:7; Ennion & Hess 2020). For physiotherapists who have already completed their community service, a three-year transitional programme (ttt-DPT) could be offered, which focuses on the same academic structure but limits the number of clinical hours/community service hours required.

The next level within the current educational sphere is a Master’s degree in physiotherapy (see Figure 2). To transition from a Master’s degree (previously obtained) to the DPT level, physiotherapists would enrol for a two-year transitional programme (tt-DPT). As in the current Master’s degree, the focus would be on research: once again, either with a research/academic (RA) or clinical (CL) focus. Within the new educational sphere (see Figure 2), no exit level is available at the Master’s degree level, mainly to enable physiotherapists to embark on a single (bigger) research project, rather than being expected to complete two research projects (i.e. one for a Master’s degree and one for a PhD/Doctorate), as pertains within the current educational sphere. This approach might encourage high-quality clinical and/or educational research and strengthen the profession and its position nationally and globally. The level of clinical and/or research/academic skills attained by physiotherapists in the proposed new educational sphere would also enable physiotherapists to take up their rightful position as **academic experts** or **clinical experts** in the South African healthcare or higher education systems and provide suitable recognition and remuneration for their particular skills.

The last, and highest, educational level in the current educational sphere is the Doctorate level, which would be equivalent to a RA/CL DPT within the new, proposed educational sphere (see Figure 2). To transition from the current doctorate level to a RA/CL DPT level, physiotherapists would be expected to enrol for a transitional programme (t-DPT), in which they should undertake research and publish at least one scholarly article, either on current or new research undertaken in the research/academic or clinical field. Previous articles would not be considered, and competence should be shown on research conducted during their enrolment for the transitional programme. The publication of an article whilst being enrolled for the transitional programme would, then, be compatible with the newly proposed RA/CL exit-level DPT programme, which would span 7 years of study (including community service). Research might also be supplemented with additional taught materials to further enhance the clinical skills of physiotherapists during their enrolment for the transitional programme. As community service is combined with the academic programme in the new educational sphere (see Figure 2), the time from entry level to exit level is shorter, requiring 1 year less of study, compared with the current educational pathway (i.e. 4-year Bachelor’s, plus 1 year of community service, plus 1 year for Master’s and 2 years for Doctorate). I believe that this progression through the academic levels would be better than in the current system, as it would all be structured, with the necessary student support structures, and should result in good throughput rates.

**FIGURE 3:**
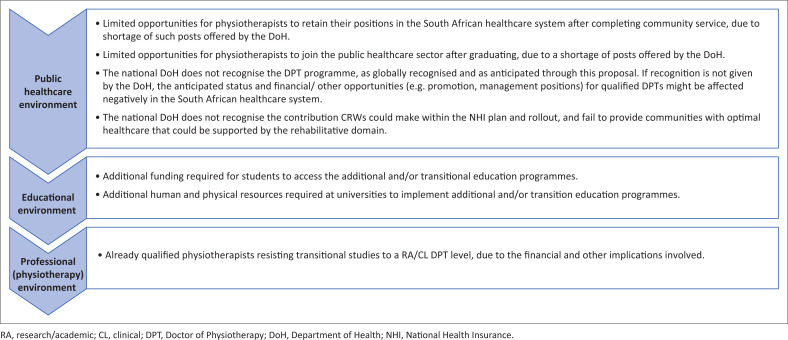
Challenges for educational transitions in Physiotherapy in the public healthcare, educational and professional environments.

However, the newly proposed educational sphere would not be without challenges. Some of the major challenges that are anticipated for the public healthcare, educational sector and/or professional sectors are included in Figure 3.

## Conclusion

My article has provided baseline suggestions for (re)considering the current educational environment for physiotherapy to ensure that the profession remains relevant and able to confront the current changes presented by the South African healthcare system, and considering the implementation of the NHI plan, whilst remaining globally aligned and competitive. The changes suggested are based on the current challenges being experienced in the physiotherapy education and healthcare service delivery arena, which could be addressed by changes in the education sphere. Based on the proposed framework, it is recommended that an in-depth feasibility study be conducted within the physiotherapy profession, focusing on aspects such as funding requirements and possibly funding options for transitional measures; analysis of new post structures within the public healthcare sector and the cost of the development, registration and approval of new educational programmes, as well as a cost analysis of educational needs, including human and physical resources.

The proposed move towards a DPT structure (see Figure 2) – which is currently taking place in the United States – could address some of the quality and skills-mix needs as envisaged in the National Health Plan 2030 and the Human Resources for Health plan. If implemented, DPT students would have to remain in the educational sector for longer, which could assist universities with clinical supervision, as well as longer-term retention of staff, which have been highlighted as challenges in the current system. It should, however, be noted that fewer graduates would be delivered to the health system in the interim period, which could influence the number of physiotherapists in the South African healthcare environment. The additional training of CRWs could, in some ways, alleviate some of the service delivery and staff shortage problems, as these mid-level workers would exit the educational sector after 2 years to enter the healthcare system (as part of the NHI) to alleviate a well-articulated service need. A shift towards the new, proposed education system might also result in more intensive profession-specific training, which could address some of the problems associated with the quality of services currently delivered in the South African healthcare sector.

Lastly, it seems that the current physiotherapy education system – that of a 4-year Bachelor/Bachelor of Science of Physiotherapy – delivers professionals who may be extremely suitable for the South African scenario. This system currently produces professionals who exit from the educational system relatively quickly as first-line practitioners (which was the strongest motivation for the United States to move towards a DPT educational system), and who are able to provide good quality healthcare services. However, because of the move in the United States to a DPT entry-level programme, and in Canada to a Master’s degree entry level, South Africa will have to ensure that its graduates remain comparable and competitive internationally. It might, thus, be realised that the optimal solution for South Africa is, indeed, one with various exit levels, as proposed in my article. Such a system could include exit levels for CRWs, who leave the education sector after a shorter time to participate in broadened service delivery, as required in the NHI system; for professional physiotherapists (as current), to provide a more general, but quality service and for DPTs with intensive, high-level profession-specific training over an extended study period, to provide expert research/academic and clinical expertise and leadership.
